# Identification of Novel Prognostic Risk Signatures of Soft Tissue Sarcoma Based on Ferroptosis-Related Genes

**DOI:** 10.3389/fonc.2021.629868

**Published:** 2021-04-06

**Authors:** Wenjing Huang, Yuhe Duan, Xiuwei Yang, Cong Shang, Xin Chen, Huanyu Zhang, Fujiang Li

**Affiliations:** ^1^ Department of Pediatric Surgery, The Affiliated Hospital of Qingdao University, Qingdao, China; ^2^ Department of Infectious Diseases, The Affiliated Hospital of Qingdao University, Qingdao, China

**Keywords:** ferroptosis, soft tissue sarcoma, immune microenvironment, nomogram, signature

## Abstract

**Background:**

The role of ferroptosis in tumorigenesis has been confirmed in previous studies. However, the comprehensive analysis of ferroptosis-related gene (FRG) to study the role of FRG in soft tissue sarcoma (STS) is lacking.

**Methods:**

RNA sequencing profile of TCGA-SARC cohort and GTEx were used to select differentially expressed FRGs (DEFRGs). Univariate, LASSO, and multivariate Cox analyses were selected to determine overall survival (OS)- and disease-free survival (PFS)-related FRGs. Two prognostic signatures were established and validated in two independent sets from Gene Expression Omnibus (GEO). Finally, the expression of key FRGs were validated with RT-qPCR.

**Results:**

In total, 198 FRGs (90.4%) were abnormally expressed in STS. Twelve DEFRGs were incorporated in the final signatures and showed favorable discrimination in both training and validation cohorts. Patients in the different risk groups not only showed different prognosis, but also showed different infiltration of immune cells. Two nomograms combining signature and clinical variables were established and the C-indexes were 0.852 and 0.752 for the OS and DFS nomograms, respectively. Finally, the expression of NOX5, HELLS, and RPL8 were validated with RT-qPCR.

**Conclusion:**

This comprehensive analysis of the FRG landscape in STS revealed novel FRGs related to carcinogenesis and prognosis. These findings have implications for prognosis and therapeutic responses, which revealed potential prognostic biomarkers and promote precision medicine.

## Introduction

Ferroptosis, as a special kind of programmed cell death, is a process of cytological changes caused by the accumulation of iron-dependent lipid hydroperoxide (1). It is marked by the oxidative modification of phospholipid membranes, which is different from traditional apoptosis or autophagy cell death (1, 2). Recently, as the understanding of ferroptosis has increased, its complex biological functions in cancers have also been revealed, even in some chemotherapeutic resistant tumors (3–5). Studies have shown that ferroptosis can inhibit tumor growth (6) and play an important role in different cancers (7, 8). Xie et al. (9) discovered that ferroptosis caused by erastin inhibit the growth of colorectal cancer cells. And ductal pancreatic cancer cells with mutant KRAS genes are more susceptible to ferroptosis when compared with wild-type cells (10). Besides, the tumor suppressor gene p53 is closely related to the sensitivity of ferroptosis. In mice with intact p53, p53 binds to the SLC7A11 promoter region and inhibits its transcription, which is essential for the induction of ferroptosis (11). On this basis, the concept of the ferroptosis-related gene (FRG) was developed, which was closely associated with tumorigenesis. For example, direct inhibition of GPX4 leads to a high necrotic cell population in adrenocortical carcinomas cells (12). Hepatocellular carcinoma cells inhibit the effect of ferritin by regulating the expression of NRF2 or MT-1G, thereby promoting sorafenib resistance in an *in vitro* model (13). Therefore, the in-depth understanding of these genes would help to reveal the role and mechanism of ferroptosis in cancer development and therapy.

Soft tissue sarcomas (STSs) are a group of malignant malignancies originating from mesenchymal tissue, including more than fifty subtypes (14, 15). On the whole, the incidence of STS is low but it is a major malignancy in the children and adolescents (16, 17). The 5-year survival rate of STS is around 50% but plummets in advanced patients (18–20). Additionally, nearly half of the STS would occur distant recurrence, which leads it hard to choose the optimal therapy, such as surgery resection, adjuvant chemotherapy and radiotherapy, or tumor immunotherapy (21). Therefore, accurate biomarkers to stratify STS patients into different risk groups and develop targeted therapies is urgent needed.

In this study, we integrated the genomic and clinical information of STS samples and comprehensively evaluated their FRGs expression. In addition to study the prognosis of STS patients, we also investigated that the relationship between FRGs and the characteristics of immune cell infiltration in STS patients.

## Materials and Methods

### Data Collection

RNA sequencing profile of the TCGA-SARC cohort and the GTEx cohort were downloaded from the UCSC browser (https://xenabrowser.net). For both datasets, RNA sequencing data (FPKM values) were normalized into log_2_(FPKM+1). The corresponding clinical data of TCGA-SARC cohort were downloaded from the cBioPortal (http://www.cbioportal.org/). In this cohort, there are 259 STS patients, including 104 with leiomyosarcomas, 58 with dedifferentiated liposarcomas, 51 with undifferentiated pleomorphic sarcomas, 25 with myxofibrosarcomas, 10 with synovial sarcomas, and 11 with other STS types. For GTEx cohort, the RNA sequencing profile of 911 normal soft tissues were downloaded to match TCGA-SARC and determine abnormally expressed genes. Moreover, we downloaded the gene expression profiling and clinical data of GSE63157 and GSE30929 to form the independent validation cohorts.

### Identification of Tumor-Related FRGs and Functional Annotation

The list of FRGs was obtained from the published literature and FerrDb database (22, 23). The differential analysis was performed between 259 tumor tissues and 911 normal tissues with the “limma” R package. FRGs with a false discovery rate (FDR) < 0.05 were considered as differentially expressed FRGs (DEFRGs). To explore the potential function of the identified DEFRGs, Gene Ontology (GO) functional annotation and Kyoto Encyclopedia of Genes and Genomes (KEGG) pathway enrichment analyses were performed in the R software with the “clusterprofiler” package (24). The results with an adjusted p-value <0.05 were considered as statistically significant.

### Construction of Prognostic FRG Signatures

Then, STS patients with follow-up data, including overall survival (OS) and disease-free survival (DFS), were incorporated into the survival analyses and construct the prognostic signatures. The establishment of the prognostic models includes the following steps. First, the univariate Cox analysis was performed, and DEFRGs with a p-value < 0.05 were considerate as OS- or DFS-related DEFRGs. To further minimize the probability of overfitting, the LASSO regression analysis was applied and significant DEFRGs were further incorporated in the multivariate Cox analysis to construct two novel FRG signatures. The coefficients of all DEFRGs in the final signature were confirmed simultaneously and were used to calculate risk scores for all STS patients. The risk score was calculated as follows:

$$Risk\;\text{Score}=\sum_{i=0}^{n}\beta_{i}*{\text{G}}_{i}$$

*β_i_* is the coefficient of the gene *i* in the multivariate Cox analysis; G*_i_* is the expression value of gene *i*; and *n* is the number of genes in the signature.

To assess the discrimination of FRG signatures, the “timeROC” package was used to generate receiver operating characteristic (ROC) curves at 1-, 2-, and 3-years, and the corresponding time-dependent area under the curves (AUCs) were calculated simultaneously. Furthermore, all patients were divided into low- and high-risk groups according to the median of risk score. Kaplan-Meier (K-M) survival curve with the log-rank test was generated to confirm the difference of prognosis between the two groups.

### Validation of the Prognostic Signatures

External validation is critical for prognostic signatures. In our research, the GSE63157 cohort was used to validate the OS signature and the GSE30929 cohort was used to validate the DFS signature. The expression profile of the genes included in the corresponding final signature were extracted and substituted into the equations for risk score calculation. All patients in the validation set were divided into high- or low-risk groups. The prediction accuracy of signatures in the validation cohorts was evaluated by ROC curve and K-M survival analysis.

### Comparison of Immune Cell Infiltration Between Different Risk Groups

In the enrichment analyses, we find that DEFRG were enriched in the immune-related pathways. A large number of studies have also shown that ferroptosis is closely related to tumor immunity (25–27). Therefore, we further studied the pattern of immune cell infiltration between different risk groups. The infiltration data of 24 immune cell infiltration data were obtained with ImmuCellAI algorithms (28). The difference of immune cell infiltration between low- and high-risk groups was confirmed by Mann-Whitney U test.

### Development of Nomograms Integrating FRG Signature and Clinical Data

Clinical data, including age, race, sex, tumor site, margin status, metastatic status, and radiotherapy, were obtained from the cBioPortal database (http://www.cbioportal.org/). The univariate Cox analysis was performed in the TCGA-SARC cohort. Clinical variables with a p value<0.05 and FRG signature were incorporated into the multivariate Cox analyses to select the independent prognostic variables. Next, two prognostic nomograms were established by the “rms” package in “R” based on the corresponding independent prognostic factors. The concordance index (C-index) was used to assess the discrimination of two nomograms, and calibration curves were generated to evaluate the concordance between actual and nomogram-predicted outcomes.

### Cell Lines and Cell Culture

The human fibrosarcoma cell line HT-1080, synovial sarcoma cell line (SW-982) and human skin fibroblast cell line (HSF) were purchased from iCell Bioscience Inc (Shanghai, China). HT-1080 cell was cultured in minimum essential medium (iCell Bioscience Inc, Shanghai, China) supplemented with 50µg/mL streptomycin (Solarbio, Beijing, China), 50U/mL penicillin (Solarbio, Beijing, China), and 10% fetal bovine serum (FBS) (Biological Industries, Israel). SW-982 cell was cultured in Leibovitz’s L15 (iCell Bioscience Inc, Shanghai, China) supplemented with 50µg/mL streptomycin (Solarbio, Beijing, China), 50U/mL penicillin (Solarbio, Beijing, China), and 10% FBS (Biological Industries, Israel). HSF cells was cultured in complete growth medium for primary fibroblast (iCell-0051a-001b, Shanghai, China). HT-1080 and HSF cells were cultured with 37°C in a humidified 5% CO_2_ incubator (ThermoFisher Scientific, USA), while the SW-982 cells was cultured with 37°C without CO_2_ incubotor (ThermoFisher Scientific, USA).

### Real-Time Quantitative PCR (RT-qPCR)

Total RNA were isolated from cells using the AG RNAex Pro Reagent (AG21101, Accurate Biotechnology, Hunan, China) according to the manufacturer’s protocol. Reverse transcription was conducted using the Evo M-MLV RT Kit with gDNA Clean for qPCR II (AG11711, Accurate Biotechnology, Hunan, China), and cDNA was used as the template in real-time fluorescence quantification. RT-qPCR was performed with the SYBR Green Premix Pro Taq HS qPCR Kit (AG11701, Accurate Biotechnology, Hunan, China) on a Real-Time PCR Detection System (Roche 480II). Independent experiments were conducted in triplicate, and β-actin served as an internal control. The following primers (Ruibiotech, Inc., Beijing, China) were used:

RPL8:
F 5’-AGAAGACCCGTGTGAAGCTG-3’
R 5’-GGTTTGTCAATTCGGCCACC-3’
NOX5:
F 5’-CCTGAAGGCTGTAGAGGCAC-3’
R 5’-TCGCTCTGCAAAGAAGGACT-3’
HELLS:
F 5’-ACACTGCTGTGATTACCCCG-3’
R 5’-AGACATGCGAGCCTTTTCCA-3’

## Results

### Patient Characteristics

**Figure 1:**
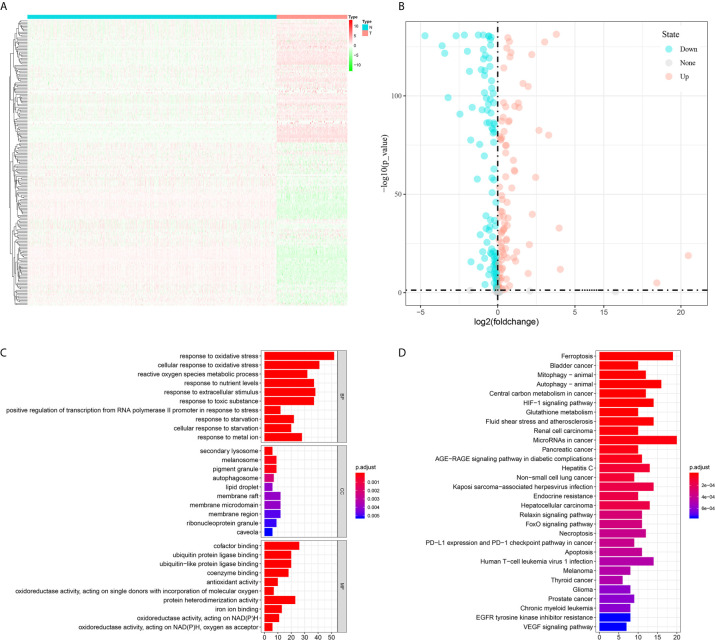
Differential analysis of ferroptosis-related genes between tumor and normal tissues and enrichment analyses of differential expressed ferroptosis-related genes. **(A)** A heatmap to show the expression of differential expressed ferroptosis-related genes in tumor and normal tissues; **(B)** A volcano plot to show the results of differential analysis; **(C)** Gene Ontology; **(D)** Kyoto Encyclopedia of Genes and Genomes.

In total, the expression profiles of 219 FRGs were obtained from both TCGA-SARC and GTEx cohorts. In these genes, 198 FRGs (90.4%) were abnormally expressed in STS, including 95 upregulated and 103 downregulated genes (**Figures 1A, B**). Then, GO and KEGG analyses were performed to annotate 198 DEFRGs. GO analysis suggested that DEFRGs were mainly enriched in response to oxidative stress, secondary lysosome, and cofactor binding in biological process, cellular component, and molecular function (**Figure 1C**). The top five sections of KEGG analysis for DEFRGs were ferroptosis, bladder cancer, mitophagy-animal, autophagy-animal, and central carbon metabolism in cancer (**Figure 1D**). These results further confirmed that 198 DEFRGs play a vital role in tumorigenesis and several important physiological processes. Intriguingly, immune-related pathways were also enriched, including PD−L1 expression and PD−1 checkpoint pathway in cancer, and Human T−cell leukemia virus 1 infection.

### Construction of Two Prognostic Signatures in the TCGA Cohort

**Figure 2:**
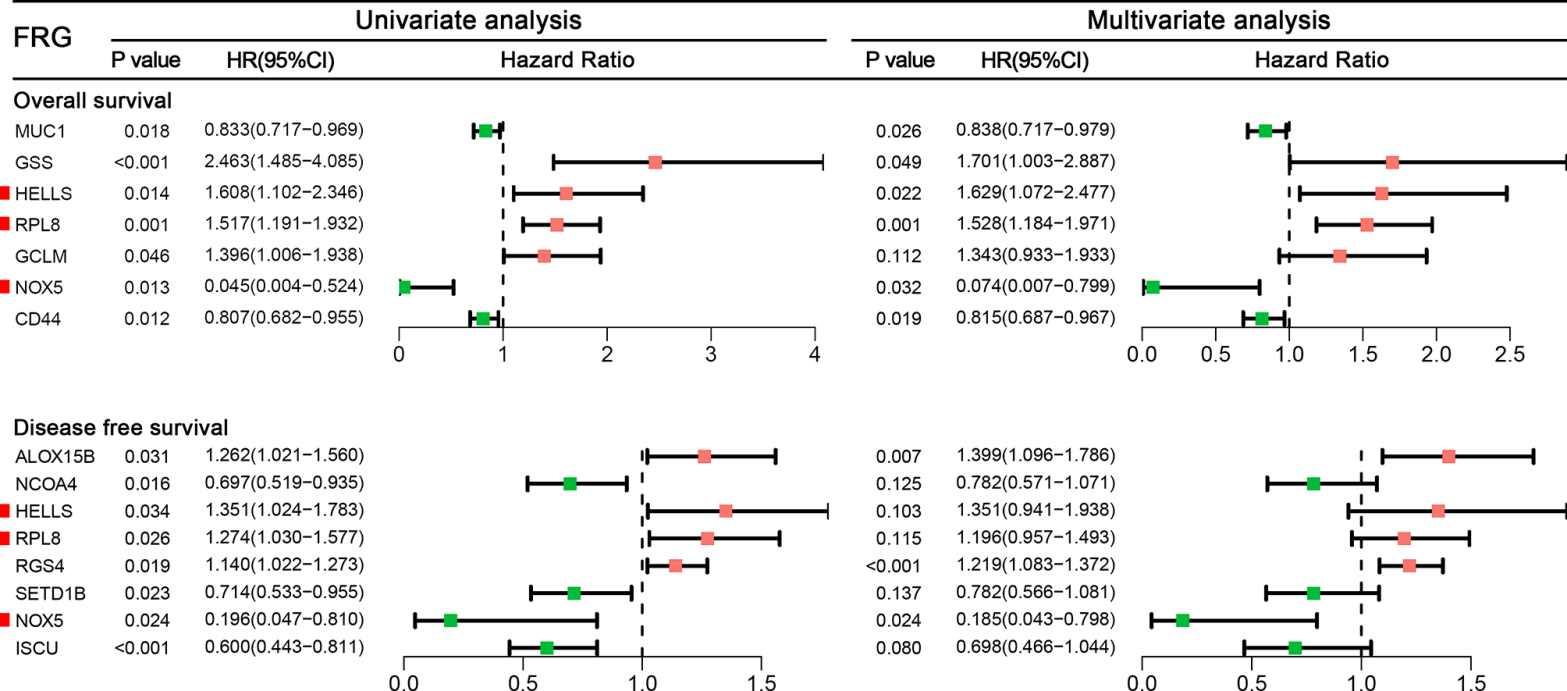
A forest to show the results of univariate and multivariate Cox analyses results for ferroptosis-related genes incorporated into the final signatures.

**Figure 3:**
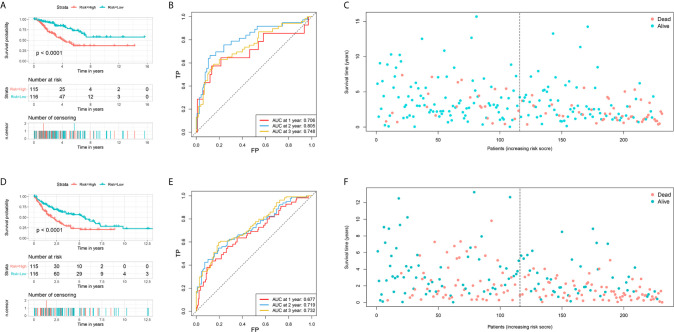
Establishment of two ferroptosis-related genes based signature for predicting the overall survival and disease-free survival in soft tissue sarcoma patients. **(A)** The survival curve shows the distinct overall survival between low- and high-risk groups; **(B)** The receiver characteristic curves of ferroptosis-related signature for predicting 1-, 2-, and 3-year overall survival; **(C)** The survival plot shows the follow up time and overall survival status; **(D)** The survival curve shows the distinct disease-free survival between low- and high-risk groups; **(E)** The receiver characteristic curves of ferroptosis-related signature for predicting 1-, 2-, and 3-year disease-free survival; **(F)** The survival plot shows the follow up time and disease-free survival status.

Among 259 STS patients, 28 patients had no DFS data. Therefore, 231 STS patients were incorporated into the survival analysis and form as the training cohort. Totally, 31 DEFRGs were confirmed as OS-related biomarkers and 22 DEFRGs were confirmed as DFS-related biomarkers. Then, 17 OS-related DEFRGs and 8 DFS-related DEFRGs were excluded from the LASSO analysis (**Supplementary Figure S1**). Furthermore, multivariate Cox analysis was performed, and 12 DEFRGs were selected to construct two prognostic signatures, including four DEFRGs for the OS signature only, five DEFRGs for the DFS signature only, and three overlapping DEFRGs (**Figure 2**). The equation of FRG signature for OS was shown as follows: Risk score=MUC1*-0.177+GSS*0.531+HELLS*0.488+RPL8*0.424+GCLM*0.295+NOX5*-2.598+CD44*-0.205. The equation of FRG signature for DS was shown as follows: Risk score= ALOX15B*0.336+NCOA4*-0.246+HELLS*0.301+RPL8*0.179+RGS4*0.198+SETD1B*-0.246+NOX5*-1.687+ISCU*-0.360. According to the corresponding median risk score, the patients were stratified into a low-risk group (n=116) or a high-risk group (n=115). The K-M survival curve showed that high-risk patients had a significantly worse OS and DFS than their low-risk counterparts (**Figures 3A, D**). In addition, ROC curves confirmed favorable discrimination of FRG signatures (**Figures 3B, E**). The AUC values of OS FRG signature for predicting 1-, 2-, and 3-year OS were 0.706, 0.805, and 0.748, respectively (**Figure 3B**). The AUC values of DFS FRG signature for predicting 1-, 2-, and 3-year DFS were 0.677, 0.719, and 0.732, respectively (**Figure 3E**). Furthermore, we can also observe a clear trend in the survival plots (**Figures 3C, F**). In other words, with the increasing risk score, the OS rate or DFS rate of the patients decreasing, and the OS time or DFS time gradually decreased (**Figures 3C, F**).

### Validation of Two FRG Signatures in GEO Datasets

**Figure 4:**
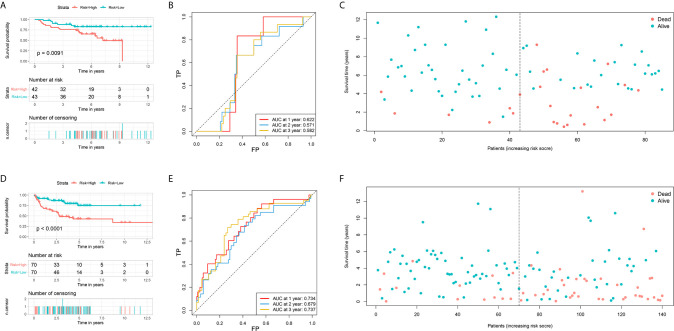
Validation of two ferroptosis-related genes based signature for predicting the overall survival and disease-free survival in soft tissue sarcoma patients. **(A)** The survival curve shows the distinct overall survival between low- and high-risk groups; **(B)** The receiver characteristic curves of ferroptosis-related signature for predicting 1-, 2-, and 3-year overall survival; **(C)** The survival plot shows the follow up time and overall survival status; **(D)** The survival curve shows the distinct disease-free survival between low- and high-risk groups; **(E)** The receiver characteristic curves of ferroptosis-related signature for predicting 1-, 2-, and 3-year disease-free survival; **(F)** The survival plot shows the follow up time and disease-free survival status.

The prognostic values of the risk score of STS patients in the validation cohort were calculated. The prognosis, including OS and DFS, of high-risk patients, were significantly worse than low-risk patients (**Figures 4A, C–F**). The AUC values of signature to predict the OS at 1-, 2-, and 3-year were 0.622, 0.571, and 0.582, respectively (**Figure 4B**). The AUC values of signature to predict the DFS at 1-, 2-, and 3-year were 0.734, 0.679, and 0.737, respectively (**Figure 4D**). Generally, two FRG signatures showed satisfactory performance in the independent cohorts, which indicated that these signatures are robust prognostic biomarkers.

### Two Risk Groups Showed Distinct Immune Cell Infiltration Patterns

**Figure 5:**
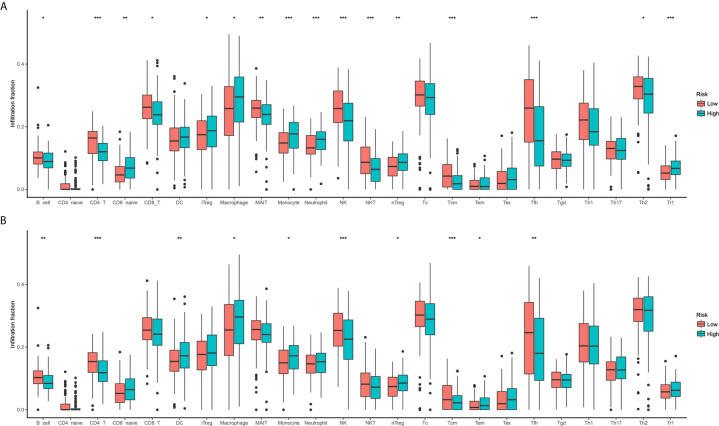
Comparison of immune cell infiltration between two risk groups. **(A)** Overall survival; **(B)** Disease-free survival.

According to the enrichment analyses, ferroptosis seems to have some interaction with the immune feature. Therefore, we further study the immune cell infiltration patterns between distinct risk groups. The immune cell infiltration in different risk groups are shown in **Figure 5**. The fractions of 16 immune cells are significantly different between two OS risk groups (**Figure 5A**), and 10 immune cells are significantly different between two DFS risk groups (**Figure 5B**). Totally, eight immune cells showed significantly different between OS subgroups and DFS subgroups. In general, the modification of immune cell infiltration may be one of the mechanisms by which ferroptosis regulates tumor progression. However, further cellular mechanisms and functional studies are needed to confirm this conclusion.

### Development of Two FRG-Clinical Nomograms to Predict the Individual Outcomes of STS Patients

**Figure 6:**
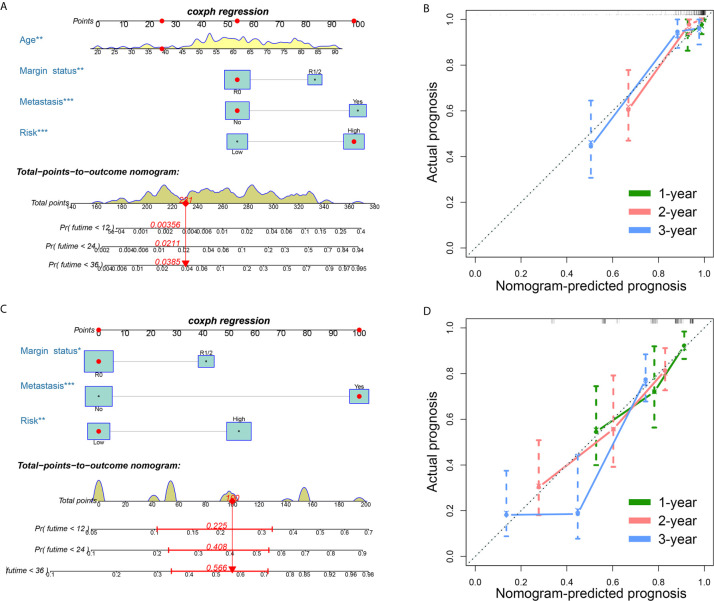
Establishment of ferroptosis-related genes-clinical nomograms for soft tissue sarcoma patients. **(A)** The nomogram for predicting overall survival of soft tissue sarcoma patients; **(B)** The calibration curves to evaluate the overall survival nomogram; **(C)** The nomogram for predicting disease-free survival of soft tissue sarcoma patients; **(D)** The calibration curves to evaluate the disease-free survival nomogram.

Clinical variables are important prognostic factors for tumor patients. Therefore, it is important to study that FRG signatures can independently predict the prognosis of STS patients. Univariate Cox analysis indicated that age, metastatic status, margin status, and multifocal indicator are OS-related factors, and metastatic status, margin status, and multifocal indicator are DFS-related factors (**Table 1**). Then, the FRG signature and corresponding significant factors in the univariate Cox analysis were incorporated into the multivariate Cox analysis. Age, margin status, metastatic status, and FRG signature were confirmed as independent OS-related factors (**Table 2**). In addition, margin status, metastatic status, and FRG signature were confirmed as independent DFS-related factors (**Table 2**). Afterward, we developed two FRG-clinical nomograms to predict the OS and DFS, respectively (**Figures 6A, C**). The C-index values of OS and DFS nomograms were 0.852 and 0.752, respectively. Additionally, calibration curves indicated that the nomogram-predicted prognosis was satisfactorily consistent with actual outcomes (**Figures 6B, D**).

**Table 1:** Univariate cox analysis for soft tissue sarcoma.

	Overall survival			Disease-free survival		
	HR	95%CI	P value	HR	95%CI	P value
Risk						
Low						
High	2.890	1.747-4.782	0.000	2.053	1.429-2.949	0.000
Age	1.020	1.001-1.038	0.035	1.010	0.998-1.023	0.113
Sex						
Female						
Male	0.903	0.563-1.448	0.672	1.090	0.767-1.548	0.632
Race						
Asian						
Black	0.713	0.082-6.219	0.759	2.122	0.264-17.028	0.479
White	0.537	0.073-3.974	0.543	1.913	0.266-13.781	0.520
Histological type						
Dedifferentiated liposarcoma						
Leiomyosarcoma	0.774	0.435-1.377	0.383	0.796	0.512-1.238	0.311
Myxofibrosarcoma	0.686	0.288-1.633	0.394	0.735	0.378-1.430	0.365
Other	0.678	0.252-1.818	0.440	0.702	0.345-1.430	0.330
Undifferentiated pleomorphic sarcoma	0.664	0.300-1.470	0.312	0.775	0.439-1.369	0.381
Metastasis						
No						
Yes	4.878	2.550-9.329	0.000	4.937	3.140-7.762	0.000
Radiotherapy						
No						
Yes	1.255	0.723-2.176	0.419	1.169	0.784-1.744	0.444
Margin status						
R0						
R1-2	2.328	1.396-3.883	0.001	2.085	1.422-3.056	0.000
Tumor site						
Extremity						
Other	1.160	0.695-1.936	0.570	0.974	0.6731.410	0.891
Multifocal indicator						
No						
Yes	2.605	1.503-4.516	0.001	2.081	1.313-3.298	0.002

HR, hazard ratio; CI, confidence interval.

**Table 2:** Multivariate cox analysis for soft tissue sarcoma.

	Overall survival			Disease-free survival		
	HR	95%CI	P value	HR	95%CI	P value
Risk						
Low						
High	5.122	2.256-11.632	<0.001	2.409	1.457-3.981	0.001
Age	1.047	1.018-1.077	0.001			
Metastasis						
No						
Yes	5.202	2.465-10.978	<0.001	4.580	2.782-7.540	<0.001
Margin status						
R0						
R1-2	2.929	1.402-6.118	0.004	1.776	1.056-2.986	0.030
Multifocal indicator						
No						
Yes	0.826	0.309-2.209	0.704	1.811	0.867-3.782	0.114

HR, hazard ratio; CI, confidence interval.

### Validation of Expression Level of Three Hub Genes in STS Cell

**Figure 7:**
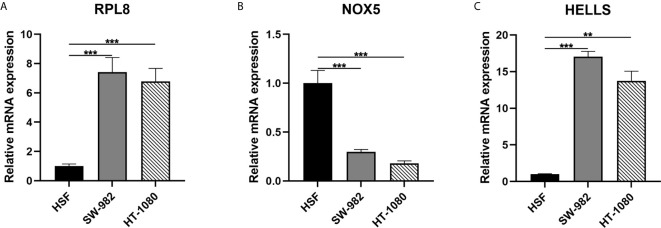
Validation of mRNA expression of three key ferroptosis-related genes in cell lines. **(A)** RPL8; **(B) **NOX5; **(C)** HELLS.

In the final signatures, 12 genes were included. Three genes, including HELLS, RPL8, and NOX5, were incorporated into both signatures (**Figure 2**). HELLS and RPL8 are risk genes for STS, while NOX5 is a protective gene. Additionally, the expression level of HELLS and RPL8 in STS sample were significantly higher than normal tissue, while the expression level of NOX5 was significantly lower. To verify this results in the cell lines, RT-qPCR was employed (**Figure 7**). The expression levels of HELLS and RPL8 in HT-1080 and SW-982 cell lines were significantly higher than HSF cell lines. Moreover, the expression levels of NOX5 was significantly lower in HT-1080 and SW-982 cell lines. The cell experiment further verified the reliability of bioinformatics results.

## Discussion

Targeted induction of cancer cell death is currently the most effective anti-cancer treatment. Recent evidence indicates that ferroptosis is an essential process in tumorigenesis and cancer treatment (29–31). However, its role in STS is still unclear. In the present study, we found that most of FRGs are abnormally expressed in STS tissues, and in univariate Cox regression analysis, more than half of the FRGs are related to the OS. These results indicated the significant role of ferroptosis in STS and the possibility of using these FRGs to establish a prognostic model. Then, 12 prognostic FRGs were incorporated into the final signatures, which showed favorable performance in both training and validation cohorts. To our knowledge, it is the first comprehensive analysis to study the role of FRG in STS, which is important for further study to study the mechanism of FRG in STS.

The prognostic model proposed in this study is composed of 12 FRGs (MUC1, GSS, HELLS, RPL8, GCLM, NOX5, CD44, ALOX15B, NCOA4, RGS4, SETD1B, ISCU). And three FRGs were included in both OS and DFS signatures. HELLS is a gene whose transcription is controlled by the RB/E2F pathway, which is believed to be the cause of epigenetic changes in retinoblastoma and is necessary for tumor production (32, 33). The results were consistent with present study. As for another gene, NOX5 showed the role of tumor suppressor genes in STS, which was lower expressed in tumors and beneficial for prognosis. However, NOX5 was confirmed to promote the proliferation of some tumor cells, such as breast cancer (34). Therefore, it is potential that NOX5 has a dual effect on cancers, which needs to be verified with further research. Besides, mucin 1 (MUC1), a tumor driver gene in our study, is a membrane-bound protein whose gene expression is highest in the respiratory, digestive, and reproductive systems, and plays a role in cell growth, differentiation, and cell signal transduction (35–37). In oral squamous cell carcinoma (OSCC), silencing MUC1 can reduce the expression of Slug, thereby inhibiting tumor cell proliferation, inhibiting DNA replication, and inducing OSCC cell apoptosis (38). In addition, MUC1 can also be used as a useful marker for predicting poor prognostic factors for 5-year survival outcome after radical esophageal squamous cell carcinoma resection (39). RGS4 was found to overexpress in glioblastoma, and its knockout reduces GSC migration, invasion, and induces apoptosis in tumor cells (40), suggesting its risk role in cancer patients like our findings. In summary, in the prognostic signature, some genes (MUC1, RGS4) have been reported to promote tumor cell growth. Moreover, in this study, the role of these genes has been confirmed and is associated with poor prognosis. However, there are few related studies on some genes, such as GSS and ISCU.

Previous studies have found that the immune mechanism plays an important role in the progression of STS, thus the immune environment is considered to be an important factor in the occurrence of STS (41). In the functional enrichment analysis of DEFRGs, some immune-related pathways and functions were detected, such as PD−L1 expression and PD−1 checkpoint pathway. Therefore, we further compared and analyzed the immune characteristics of high- and low-risk groups. From the comparison results, we can conclude that the levels of CD4 T cells and NK cells are higher in the low-risk group, indicating that these two cells may have a positive effect on the prognosis of STS. Smith et al. (42) found that the activation of NK cells is related to the prognosis of STS patients. In addition, Zhang et al. (43) found that infiltrating immune cells are related to the survival, treatment response, and prognosis of breast cancer patients, including T cells. Macrophages and monocytes are just the opposite. The levels are higher in high-risk patients, indicating that they may be used as negative predictors or risk factors for prognosis. A study has found that macrophages induce the expression of PD-L1 in tumor cells, help tumor cells escape the killing of cytotoxic T cells, thereby promoting tumor cell proliferation (44). So, our research results have been confirmed in other studies.

In the nomograms, we established, in addition to the established signatures, several common clinical variables such as patient age and tumor metastasis were also included. These other studies were also used as risk factors for STS, suggesting the prognosis of these characteristics value has been widely recognized (45, 46). After univariate and multivariate Cox regression analyses, we found that the risk score of FRG signature is also an independent risk factor affecting the prognosis of STS. Therefore, incorporating it into the establishment of the nomogram can more accurately predict the prognosis of patients. DCA and the calibration curve show the effectiveness of the nomogram. Although nomogram has been combined with clinical variables, like specific genes and other predictors to predict the prognosis of STS (47, 48), this is the first study to use FRGs to predict the prognosis of STS patients and provides new ideas for the prognosis of STS patients.

Although our research results have certain prospects, there are still some limitations. Firstly, we used different external data sets for verification, but it is still a retrospective study, which is inherently biased and lacks follow-up data. Secondly, due to the limitations of public databases, only a small number of clinical variables are available, and other variables that may affect the prognosis of patients are not included, such as specific treatment plans. Therefore, more factors need to be obtained to construct a more accurate nomogram. Finally, three cell lines but not STS and normal control in tissues were used in our research, which is also a limitation of our research.

## Conclusion

In conclusion, FRG plays an important role in STS patients and related with the progression of the tumor. Two validated FRG-related signatures were established and showed favorable prognostic value. However, further studies are needed to study the potential mechanism and validate the nomogram that developed in our present study.

## Data Availability Statement

Publicly available datasets were analyzed in this study. This data can be found here: https://xenabrowser.net/ (cohort: GDC TCGA Sarcoma and cohort: GTEX); https://www.cbioportal.org/study/summary?id=sarc_tcga; and https://www.ncbi.nlm.nih.gov/geo (GSE63157 and GSE30929).

## Author Contributions

FL, YD, and WH performed the data analysis and wrote the manuscript. XY, CS, XC, and HZ contributed to the data analysis and manuscript revision. FL and YD contributed to literature search and data extraction. WH and YD conceived and designed the study. All authors contributed to the article and approved the submitted version.

## Conflict of Interest

The authors declare that the research was conducted in the absence of any commercial or financial relationships that could be construed as a potential conflict of interest.
