# Supplementary figures and images for: Identification of Novel Prognostic Risk Signatures of Soft Tissue Sarcoma Based on Ferroptosis-Related Genes
Source: Front Oncol. 2021 Apr 6;11:629868. doi: 10.3389/fonc.2021.629868 (PMC8056866; doi:10.3389/fonc.2021.629868)

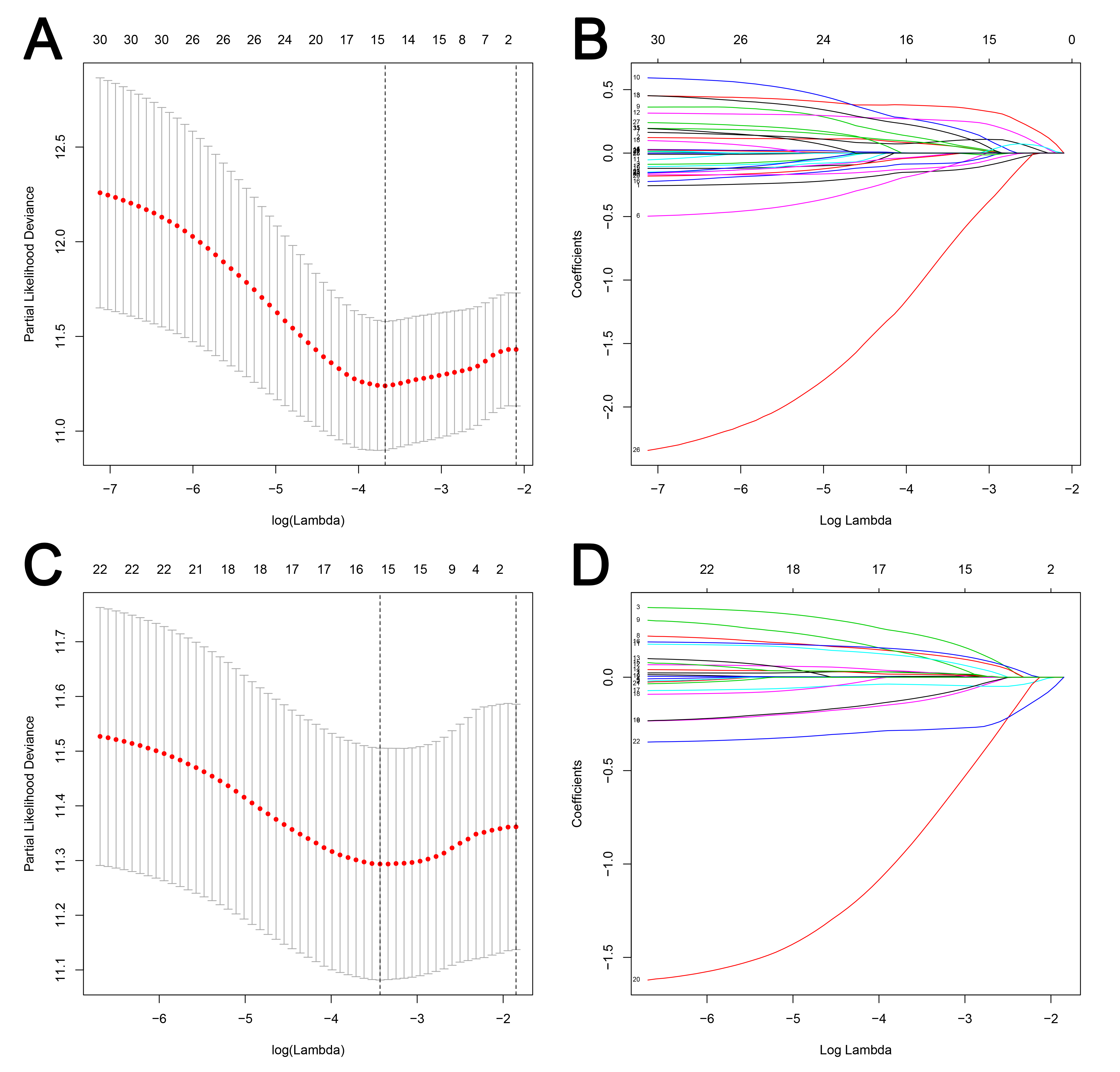

Supplement: Supplementary file 1 [file Image_1.tif]
